# Supplementary material for: Genomic and common garden approaches yield complementary results for quantifying environmental drivers of local adaptation in rubber rabbitbrush, a foundational Great Basin shrub
Source: Evol Appl. 2021 Nov 27;14(12):2881–900. doi: 10.1111/eva.13323 (PMC8674890; doi:10.1111/eva.13323)
Supplement: Supplementary file 4 — Appendix S2 [file EVA-14-2881-s001.docx]

| Variable | Used | Description |
| --- | --- | --- |
| SuppTable3 | **Yes, used blank, not used  NA, not applicable** | **Environmental Variables** |
| Name | NA | Collection location |
| Pop | NA | Two letter abbreviation |
| State | NA | State of collection location |
| Elev | Yes | Measured elevation in m from USGS DEM models |
| Variety | NA | Varietal designation with the E. nauseosa complex |
| N | NA | Number of individuals sequenced within population |
| prcpann | Yes | Total annual precip from PRISM |
| ProvisionalSeedZone |  | Provisional seed zone (cite Bower 2014 article) pulled from https://www.fs.fed.us/wwetac/threat-map/TRMSeedZoneMapper.php |
| Level3Ecoregion |  | EPA's level 3 ecoregion, used GIS shapefile taken from USGS website |
| GRSGHabitat |  | FWS's greater sage grouse habitat description, used GIS shapefile from Sarah Kulpa |
| ELELSeedZone |  | USFS's preliminary squirreltail seed zone, used GIS shapefile from Sarah Kulpa |
| ResistanceResilience |  | BLM's Resistance and resilience at description, used GIS shapefile from Sarah Kulpa |
| POSESeedZone |  | USFS's Sandberg bluegrass seed zone, pulled from https://www.fs.fed.us/wwetac/threat-map/TRMSeedZoneData.php |
| ACTHSeedZone |  | USFS's Thurber’s needlegrass seed zone, pulled from https://www.fs.fed.us/wwetac/threat-map/TRMSeedZoneData.php |
| Year |  | Used PRISM's 30-year normals from 1981-2010 |
| SLOPE | Yes | Slope in degrees, derived from USGS DEM models |
| ASPECT | Yes | Aspect in degrees measured from North, derived from USGS DEM models |
| Af |  | Folded aspect calculated as \|180 - \|aspect - 225\|\| |
| SlopRad |  | Slope converted to radians |
| AfRad |  | Folded aspect converted to radians |
| LatRad |  | Latitude converted to radians |
| HL | Yes | McCune and Keon 2002 Heat Load Index which is used as a multiplier for PET and is calculated from slope, folded aspect, and latitude |
| SoilMax | Yes | Maximum soil water holding capacity 0-150 cm (derived from USDA SSURGO data) |
| CumlAET | Yes | Sum of AET in twelve months in Redmond calculations |
| CumlCWD |  | Sum of CWD in twelve months in Redmond calculations |
| CumlPET |  | Sum of PET in twelve months in Redmond calculations |
| CumlWs |  | Sum of Wm in Redmond calculations (equals annual precip, same value) |
| AnnualMeanTemp | Yes | Mean annual temperature from PRISM |
| maxtemp |  | PRISM highest single month (mean) maximum temperature |
| mintemp |  | PRISM lowest single month (mean) maximum temperature |
| mntmpfal |  | PRISM average min temp from Sep-Nov |
| mntmpspr |  | PRISM average min temp from Mar-May |
| mntmpsu |  | PRISM average min temp from June-Aug |
| mntmpwin |  | PRISM average min temp from Dec-Feb |
| WsAETspr |  | Difference between water supply and AET during the spring |
| FallAET |  | Difference between actual evapotranspiration summer low and fall peak |
| mxtmpfal |  | PRISM average max temp from Sep-Nov |
| mxtmpspr |  | PRISM average max temp from Mar-May |
| mxtmpsum |  | PRISM average max temp from June-Aug |
| mxtmpwin |  | PRISM average max temp from Dec-Feb |
| prcpfal |  | PRISM total precip from Sep-Nov |
| prcpspr |  | PRISM total precip from Mar-May |
| prcpsum |  | PRISM total precip from June-Aug |
| prcpwin |  | PRISM total precip from Dec-Feb |
| temprang |  | PRISM maxtemp - mintemp |
| pcseas | Yes | Precipitation Seasonality Index (Walsh and Lawler, 1981) [1] |
| MeanDewpointTemp |  | PRISM mean dewpoint temp |
| MinVPD | Yes | PRISM average min vapor pressure deficit |
| MaxVPD |  | PRISM average max vapor pressure deficit |
| DeclAET |  | Steepest rate of decline of actual evapotranspiration |
| DurCWD |  | Duration of climatic water deficit during the summer |
| OnsetCWD |  | Onset of climatic water deficit during the summer |
| MaxMoCWD |  | Month of maximum of climatic water deficit |
| MaxCWD |  | Magnitude of climatic water deficit |
| AETgdd |  | Cumulative annual actual evapotranspiration during the growing season |
| CumlGDD |  | Calculation using PRISM temperature data, added up for all twelve months [2] |
| DurGDD |  | Duration of growing degree-days |

1. Precipitation Seasonality Index (Walsh and Lawler, 1981)


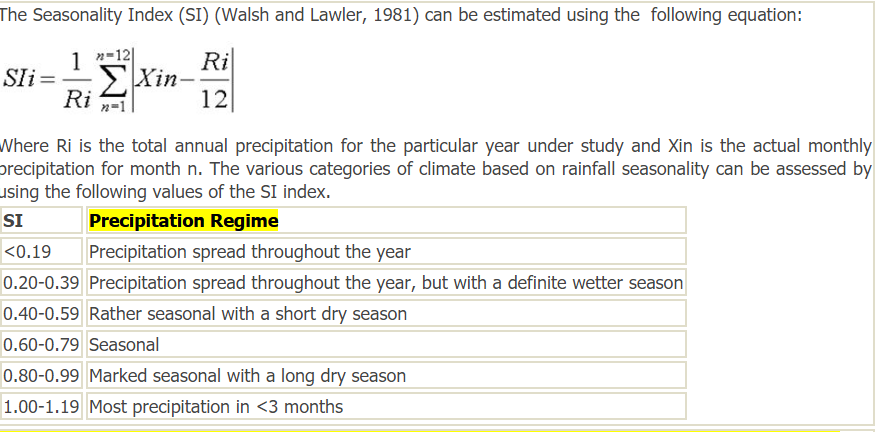


2. Growing Degree-Days (GDD) Calculation using PRISM temperature data


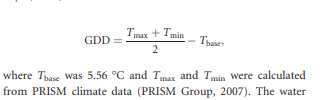


| SuppTable1 | Yes, used blank, not used  NA, not applicable | Common garden (phenotypic) measurements |
| --- | --- | --- |
| Name | NA | Collection location |
| Pop | NA | Two letter abbreviation |
| State | NA | State of collection location |
| Elev | Yes | Measured elevation in m from USGS DEM models |
| Variety | NA | Varietal designation with the E. nauseosa complex |
| N | NA | Number of individuals sequenced within population |
| Emerg | Yes | Seedling emergence, fraction of seedlings that emerged (out of 100 planted) and survived until harvest at 40 days |
| Days2Emerg | Yes | days to emergence, days from planting to emergence |
| RootBiomass |  | Mass of roots (mg) |
| ShootBiomass | Yes | Mass of shoots (mg) |
| TotalBiomass |  | Mass of roots and shoots (mg) |
| Length |  | Total length of roots, measured by WinRhizo (cm) |
| ProjArea |  | Project area, measured by WinRhizo (cm^2^) |
| SurfArea |  | Surface area, measured by WinRhizo (cm^2^) |
| AvgDiam |  | Average root diameter, measured by WinRhizo (mm) |
| LenPerVol |  | Root length per volume, measured by WinRhizo (cm/mm^3^) |
| FineRoots |  | Total root length below 0.4mm |
| CoarseRoots |  | Total root length above 0.4mm |
| FineRoots_perc |  | Percentage of total root length under 0.4mm |
| Seed | Yes | Seed weight, average weight (g) of ten batches of ten filled seeds per population |
